# Alternative RNA splicing in tumour heterogeneity, plasticity and therapy

**DOI:** 10.1242/dmm.049233

**Published:** 2022-01-11

**Authors:** Sebastian Öther-Gee Pohl, Kevin B. Myant

**Affiliations:** Cancer Research UK Edinburgh Centre, Institute of Genetics of Cancer, The University of Edinburgh, Western General Hospital, Edinburgh EH4 2XU, UK

**Keywords:** Cellular plasticity, Colorectal cancer, RNA splicing, Tumour heterogeneity

## Abstract

Alternative splicing is a process by which a single gene is able to encode multiple different protein isoforms. It is regulated by the inclusion or exclusion of introns and exons that are joined in different patterns prior to protein translation, thus enabling transcriptomic and proteomic diversity. It is now widely accepted that alternative splicing is dysregulated across nearly all cancer types. This widespread dysregulation means that nearly all cellular processes are affected – these include processes synonymous with the hallmarks of cancer – evasion of apoptosis, tissue invasion and metastasis, altered cellular metabolism, genome instability and drug resistance. Emerging evidence indicates that the dysregulation of alternative splicing also promotes a permissive environment for increased tumour heterogeneity and cellular plasticity. These are fundamental regulators of a patient's response to therapy. In this Review, we introduce the mechanisms of alternative splicing and the role of aberrant splicing in cancer, with particular focus on newfound evidence of alternative splicing promoting tumour heterogeneity, cellular plasticity and altered metabolism. We discuss recent *in vivo* models generated to study alternative splicing and the importance of these for understanding complex tumourigenic processes. Finally, we review the effects of alternative splicing on immune evasion, cell death and genome instability, and how targeting these might enhance therapeutic efficacy.

## Abstract (precis)

**Summary:** Recent evidence demonstrates that alternative mRNA splicing can influence tumour heterogeneity, its plasticity and a patient's response to therapeutic intervention. Here, we review the current knowledge of the role of alternative splicing in these processes.

## Introduction

Malignant transformation involves several cellular processes, many of which rely on aberrant expression of protein-coding genes. Most mammalian protein-coding genes contain introns that are transcribed into pre-mRNA, but excised in the process of transcript maturation. Alternative mRNA splicing (AS) involves the removal of both introns and exons from pre-mRNA transcripts, processing them into different mature mRNA variants, which, when translated, can produce proteins of alternate function. Many splicing factors are overexpressed during carcinogenesis, which increases the expression of alternative gene isoforms that can lead to the activation of many oncogenic pathways, including the Wnt (Gudino et al., 2021b), MAPK (Osada et al., 2019) and MYC (Hsu et al., 2015) pathways. Furthermore, alternative isoforms that are currently the subject of targeted immunotherapy (discussed in more detail below), such as CD19, may confer therapeutic resistance (Sotillo et al., 2015). Although it seems that AS fundamentally impairs a patient's response to therapy, recent evidence demonstrates that AS can lead to the production of neoantigens, increasing the response to immunotherapy (Lu et al., 2021). These seemingly contradictory findings highlight the extremely complex nature of AS in cancer.

Recent technological developments have fostered an increasingly complete understanding of AS (Box 1). This Review evaluates this progress, summarises how AS is tightly regulated in normal cells, and discusses how it can contribute to malignant transformation by affecting tumour cell plasticity, metabolism, genomic stability, intratumoural heterogeneity and, importantly, a patient's therapeutic response. We also discuss how aberrant AS can present a therapeutic vulnerability and highlight how research in model systems can foster successful translation of our understanding of this process into clinically viable treatments for patients.

**Box 1. Studying alternative splicing**

The quantitative detection of alternative splicing events has been fast-tracked by the increased availability of deep-sequencing platforms and reduced cost of next-generation sequencing. It has also been facilitated by the increase in computational methods for differential splicing analysis, such as SUPPA2, DiffSplice and DEXSeq. Furthermore, transcriptome-wide discovery of splicing factor-bound regulatory elements by high-throughput sequencing of RNA isolated by crosslinking immunoprecipitation (HITS-CLIP) can help directly determine the RNA-binding proteins responsible for many dysregulated events found in cancer.

These techniques have been predominantly performed *in vitro*. Transitioning from 2D cell-line models into 3D organoid culture has increased the precision with which researchers can track relevant phenotypic outcomes. In particular because various viral-based overexpression, CRISPR/Cas9-mediated knock-in, or knockdown of splice isoform genetic techniques can be applied to 3D cultures with relative ease. Finally, splice isoform-specific knockdown using anti-sense morpholinos can be easily delivered into 3D cultures for targeting of alternatively spliced isoforms and therapeutic screening.

Currently, a move to more physiologically relevant *in vivo* models is allowing researchers to observe complex phenotypes and to test therapeutic vulnerabilities, especially in cancer. Mutant splicing factor models of cancer susceptibility, like the U2AF1^S34F^ mutant knock-in mouse and isoform-specific Cre-mediated deletion models of oncogenic isoforms, can provide fundamental insights into disease aetiology and complex processes such as tumour heterogeneity and cellular plasticity.

## Mechanisms of AS

### Mechanism of exon recognition

AS predominantly occurs co-transcriptionally, but is also influenced by chromatin and epigenetic factors. It occurs in a series of steps catalysed by a large multi-ribonucleoprotein (RNP) complex, the spliceosome. The spliceosome is primarily composed of five small nuclear ribonucleoproteins (snRNPs) called U1, U2, U3, U5 and U4/6, although over 150 accessory proteins are also needed for efficient recognition of splice sites within the pre-mRNA molecule and for splicing itself (Cherry and Lynch, 2020; Wilkinson et al., 2020).

During mRNA splicing, non-coding introns are removed by two transesterification reactions, which precede the enzymatic ligation of the coding exons (Tseng and Cheng, 2013). Recognition of splicing regulatory elements, such as intronic splicing enhancers and silencers, and exonic splicing enhancers and silencers, can result in five types of AS (Sciarrillo et al., 2020):

(1) Exon skipping: exons are ‘skipped over’ and not included in the mRNA. A process that affects numerous transcripts and that can occur via multiple mechanisms, for example via mutation or transcriptional alterations in the proteins that regulate splicing or via mutations in splice donor or acceptor sites on the pre-mRNA (Anna and Monika, 2018).
(2) Mutually exclusive exons: the generation of alternative transcripts in a process in which only a single exon among multiple neighbouring ones is retained in the mature transcript (Hatje et al., 2017).
(3) Alternative 5′ splice site: an alternate donor splice site that changes the 3′ position of the upstream exon.
(4) Alternative 3′ splice site: an alternate acceptor splice site changing the 5′ position of the downstream exon.
(5) Intron retention: the non-coding intronic regions are retained in the mature mRNA.

**Fig. 1.**
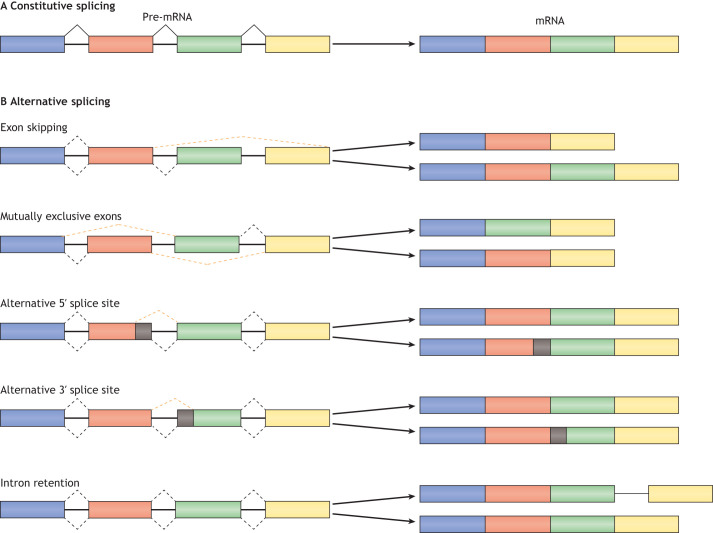
**Mechanisms of alternative splicing.** (A) Constitutive splicing excises all introns in the pre-mRNA at the correct exonic boundaries to produce a mature transcript containing all exons. (B) Alternative splicing, which alters the exon structure of the mature transcript, can occur via five separate mechanisms: (1) exon skipping – where exons are ‘skipped’ over and are thus excluded; (2) mutually exclusive exons – where only a single exon among multiple neighbouring ones is retained in the mature transcript; (3) alternative 5′ splice site – where the spliceosome uses an alternate donor splice site that changes the 3′ position of the upstream exon; (4) alternative 3′ splice site – an alternate acceptor splice site changes the 5′ position of the downstream exon; (5) intron retention – the non-coding intronic regions are retained in the mature mRNA. Adapted from Sen (2018) under the terms of the CC-BY 4.0 license.

These five different types of AS (Fig. 1) are regulated through both *cis-* and *trans-*acting elements. *Cis*-acting elements are nucleotide sequences that include splicing regulatory elements on splice sites and other regulatory regions in both exons and introns of the pre-mRNA. They can both enhance and suppress the inclusion or exclusion of exons and introns, leading to the different types of AS. These sequences are recognised by *trans*-acting elements, also known as splicing factors, and core components of the spliceosome to mediate alternatively spliced isoforms (Will and Luhrmann, 2011). *Trans*-acting elements include the serine-arginine (SR) and heterogeneous nuclear ribonucleoprotein (hnNRNP) family of proteins (Shkreta and Chabot, 2015). SR proteins predominately facilitate the recognition and inclusion of exons by the spliceosome by binding exonic splicing enhancers through their RNA recognition motifs (Long et al., 2019). HnRNPs primarily act as AS repressors, competing with SR proteins to antagonise spliceosome elements to promote exon skipping (Busch and Hertel, 2012). As mentioned above, there is growing evidence that, in addition to regulation by direct *cis*- and *trans*-acting factors, AS can also be regulated at the chromatin and epigenetic regulation levels.

Two models exist for the mechanisms by which chromatin and epigenetic modifications can influence AS. The first, known as the transcription kinetics model, posits that both nucleosome positioning on exons and histone modifications within the gene body can result in both the inclusion or exclusion of exons due to changes in the elongation rate of RNA polymerase II. For example, slower RNA polymerase II elongation can favour exon skipping by allowing increased recognition of weaker splice sites (Dujardin et al., 2014). Furthermore, RNA polymerase elongation rates and therefore pre-mRNA splicing can be regulated by intergenic histone modifications, including histone H3K9 acetylation (Schor et al., 2009) and H3K9 methylation (Schor et al., 2013). Perhaps the most striking evidence of histone modifications regulating pre-mRNA maturation is proposed in the second model, whereby histone marks recruit splicing factors to regulate AS. This has been demonstrated by Sims et al., who observed that H3K4 trimethylation marks localised in close proximity to the 5′ regions of genes recruited U2 snRNP components, including SF3A1, SF3A2 and SF3B3, in a manner dependent on the chromatin remodeller CHD1, which modulated the efficiency of pre-mRNA splicing (Sims et al., 2007). In cancer, depletion of SETD2, the major methyltransferase responsible for H3K36 trimethylation, promotes intestinal tumourigenesis by increasing Wnt signalling. SETD2 mediates intron retention in the *DVL2* transcript, which encodes a core signal transduction protein in the canonical Wnt pathway (Pohl et al., 2017), generating an isoform subjected to nonsense-mediated decay (Yuan et al., 2017). SETD2 depletion increases the expression of functional DVL2, promoting Wnt-induced transformation, stemness and intestinal regeneration (Yuan et al., 2017). Thus, chromatin-mediated alterations in RNA splicing can directly modulate complex physiological processes such as intestinal stem cell function and tumourigenesis. These studies also highlight the complex regulatory mechanisms of AS.

## Aberrant splicing in cancer

### Overview of dysregulated splicing in cancer

It is widely recognised that AS is a key cellular process dysregulated in many cancer types. Hotspot and loss-of-function (LoF) mutations in genes across the A, C and U2 complex, SR proteins and hnRNPs contribute significantly to tumourigenesis. A recent study across 33 cancer types identified 119 mutations in splicing factor-associated genes that could be classified as likely drivers (Seiler et al., 2018a). The predominant hotspot mutations were found in genes encoding the core U2 complex components SF3B1 and U2AF1, as well as the splicing factor SRSF2, whereas the prevailing LoF mutations were found in the gene encoding far-upstream binding protein 1 (FUBP1). These mutations were responsible for driving diverse splicing alterations. For example, the *SF3B1*^K700E^ mutation disrupts canonical recognition of the intronic branch point sequence, leading to alternative 3′ splice site selection (Darman et al., 2015; Zhang et al., 2019). One consequence of this mutation is increased Notch pathway activity due to the emergence of an alternatively spliced DVL2 variant (Wang et al., 2016a). Commonly found mutations in *U2AF1* cause abnormal exon inclusion through the use of a consensus sequence adjacent to its usual AG dinucleotide-interacting sequence at various 3′ splice sites (Ilagan et al., 2015; Shirai et al., 2015). The *U2AF1*^S34F^ mutation, prevalent in myelodysplastic syndrome (MDS), has also been studied through a conditional knock-in mutant mouse model. Introduction of the mutant allele profoundly changed global AS, and, in particular, in *Gnas*, *Kdm6a* and *Rac1* genes. This resulted in myeloid pathologies including macrocytic anaemia and multilineage cytopaenia, as well as a significant reduction in haematopoietic stem cells – all mirroring the human MDS presentation (Fei et al., 2018). Furthermore, recent evidence has implicated recurrent mutations in the U12 family member ZRSR2 in impaired minor intron excision-driven haematopoietic malignancies. This occurred specifically through LZTR1 minor intron retention, which was also found to exhibit intronic mutations at branch points in human cohorts of the genetic disorder Noonan syndrome (Inoue et al., 2021). Thus, mutations in the key residues that splicing factors use to recognise specific pre-mRNA sequences can lead to pathologic AS events implicated in various human malignancies.

Although comprehensive analyses and summaries of somatic mutations in splice factor genes can be found elsewhere (Seiler et al., 2018a; Wang and Aifantis, 2020), it should also be noted that general changes in the levels of expression of splicing factors can also contribute to human disease. For example, the overexpression of multiple splicing factors has been implicated in mammary gland transformation and breast cancer metastasis (Park et al., 2019). In addition, the splicing factors TRA2β and SRSF1 are direct transcriptional targets of oncogenic MYC expression. Thus, commonly upregulated pro-oncogenic pathways can mediate splicing factor overexpression, leading to malignant transformation (Park et al., 2019). Changes in AS have been linked to the majority of classical cancer hallmarks such as proliferation, apoptosis and invasion. Therefore, it is perhaps unsurprising that emerging evidence has also linked the dysregulation of AS to more recently described oncogenic phenomena. Next, we discuss these newly discovered links, focusing on tumour heterogeneity, cellular plasticity and tumour cell metabolism, and outline the development of new *in vivo* models required to study such complex processes.

### Evidence for AS in tumour heterogeneity

**Fig. 2.**
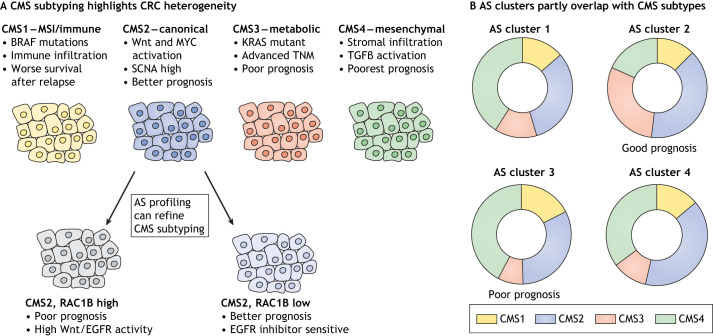
**Role of AS in tumour heterogeneity.** (A) AS can be used to differentiate CRC subtypes and these partially overlap with transcriptional subtypes, such as the widely used CMS. Among the AS subtypes, CMS3 features advanced TNM stage and is predictive of poor prognosis and thus overlaps with CMS4. In addition, AS can be used to further refine the CMS subtypes, as has been found for expression of the RAC1B splice variant, which marks a poor-prognosis subgroup of CMS2 tumours. (B) AS can also be used for molecular subtyping. In CRC, the phenotypic characteristics of AS clusters partly overlap with those of the CMSs, in particular AS cluster 3 with the poor-prognosis CMS4. Figure generated based on the results described by Xiong et al. (2018). AS, alternative mRNA splicing; EGFR, epidermal growth factor receptor; CMS, consensus molecular subtype; CRC, colorectal cancer; MSI, microsatellite instable; SCNA, somatic copy number alterations; TNM, tumour, node and metastasis.

Tumour heterogeneity refers to the presence of different cellular subpopulations with differing molecular characteristics and phenotypes within tumours. This heterogeneity can be within the same tumour or between different tumours from the same tissue. Recognition of the roles inter- and intra-tumour heterogeneity play in cancer development has led to the identification of molecular subtypes of various cancer types based on genomic, transcriptomic and proteomic data. This has aided patient genomic and transcriptomic stratification, which has improved the clinical efficacy of targeted treatments. A primary example of this is the stratification of colorectal cancer (CRC) into four consensus molecular subtypes (CMSs; Fig. 2A) classified by their gene expression signatures, copy-number alterations and mutational profiles, which have important consequences for treatment options and patient prognoses (Guinney et al., 2015). Intra-tumour heterogeneity may also promote drug resistance and relapse by selective expansion of subpopulations that may be resistant to therapies (Dentro et al., 2021). As one of the primary functions of AS is to generate proteomic and transcriptomic diversity, its link to both cellular and molecular tumour heterogeneity and the therapeutic consequences of this have started to emerge. The expression of alternatively spliced isoforms can be used to identify tumour AS subclusters and predict patient survival. In CRC, these AS clusters showed some overlap with the above-mentioned CMSs, in particular the poor prognosis-associated CMS4 (Xiong et al., 2018) (Fig. 2B). Additionally, our own group recently identified splicing of *RAC1B* as a marker of a distinct subgroup of patients with CMS2 CRC with high levels of Wnt activity and poor prognosis (Gudino et al., 2021b). Together, this indicates that transcriptional and splicing processes are closely linked, and suggests that AS-based subclustering could be a useful tool for fine-tuning the predictive power of gene expression-based tumour subtyping (Fig. 2). Interestingly, in a cohort of pancreatic ductal adenocarcinomas tissue samples, subclustering based on overall gene expression levels was not a robust predictor of patient survival. However, subclusters based on differences in intron retention events were found to be better biomarkers and survival predictors (Tan et al., 2020a). This exemplifies the importance of AS in distinguishing tumour subclusters, which may be important biomarkers in disease progression. Furthermore, AS can be explored at the single-cell level to identify heterogeneous cell types of the tumour microenvironment. A study examining 5063T cells from hepatocellular carcinoma (HCC) identified 1176 differentially expressed splicing junctions between 11 T-cell clusters. This study found preserved AS patterns between functionally similar cells, although perhaps the most interesting finding was the discovery of functional subpopulations that could be identified by alternative isoforms of a single gene, *ARHGAP15*, which encodes a RAC1-specific GTPase-activating protein (Liu et al., 2021). This finding indicates that AS analysis of cells within the tumour microenvironment can reveal cellular population heterogeneity and functional subgroups. The use of single-cell technologies in combination with deep sequencing to reveal variations in alternatively spliced isoforms is an emerging field and may have the power to reveal new biomarkers and functional cell subpopulations, with valuable prognostic implications.

### AS in cellular plasticity

Cellular plasticity, which is the ability of cells to shift phenotypic state, is critical for driving tumourigenesis and tumour progression. The best-studied form of cellular plasticity described in cancer is trans-differentiation, by which a tumour cell converts from one phenotypic type to another. The function of AS in this process will be described in the following section.

#### Trans-differentiation

**Fig. 3.**
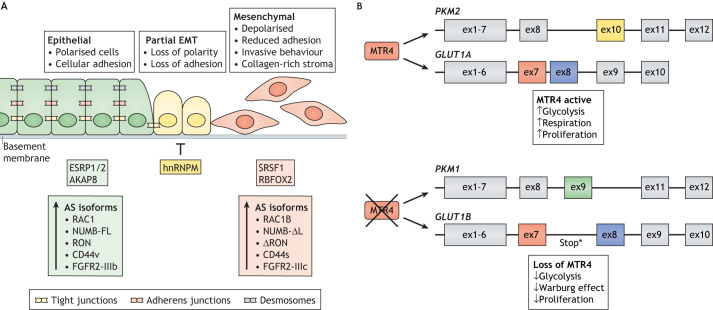
**Role of AS in tumour cell plasticity.** (A) EMT is marked by changes in cell polarity, adhesion, invasive potential and stromal composition. The splicing factors ESRP1 and AKAP8 define epithelial phenotypes, whereas hnRNPM, SRSF1 and RBFOX2 control mesenchymal phenotypes. Numerous AS events are critical in mediating the key phenotypic transitions, including EMT TF activation, loss of cell polarity, invasiveness and ECM interactions. (B) AS can also affect cancer metabolism. The RNA helicase MTR4 is central to this process, as it controls the splicing of pyruvate kinase and the glucose transporter GLUT1. The transcript for pyruvate kinase can be alternatively spliced through mutually exclusive exons. Inclusion of exon 9 results in the PKM1 isoform, whereas inclusion of exon10 generates PKM2. PKM1 increases cellular respiration. The *GLUT1* mRNA also undergoes AS to include part of intron 7, generating the GLUT1B isoform. MTR4 promotes the expression of PKM2 and GLUT1A isoforms, whereas loss of MTR4 increases PKM1 and GLUT1B expression, resulting in decreased glycolytic function and the Warburg effect in cancer cells and decreased proliferation. Figure generated based on the results described by Yu et al. (2020). AKAP8, A-kinase anchor protein 8; EMT, epithelial-to-mesenchymal transition; ESRP1, epithelial splicing regulatory protein 1; hnRNPM, heterogeneous nuclear ribonucleoprotein M; MTR4, ATP-dependent RNA helicase DOB1; PKM, pyruvate kinase; RBFOX2, RNA-binding protein fox-1 homolog 2; SRSF1, serine/arginine-rich splicing factor 1; TF, transcription factor.

The most commonly recognised form of tumour cell trans-differentiation is epithelial-to-mesenchymal transition (EMT), whereby epithelial cells lose polarity and cell–cell adhesion properties and acquire a mesenchymal phenotype. This phenotypic shift is characterised by loss of E-cadherin and other epithelial markers, elevated levels of mesenchymal cell markers such as vimentin and α-SMA, stromal remodelling and by activation of Wnt signalling and transcription factors such as the Twist proteins, Snail (also known as SNAI1) and Slug (also known as SNAI2). This process is believed to be important for allowing the migration and invasion of tumour cells and the initiation of metastasis (Kalluri and Weinberg, 2009). AS is extensively linked to EMT, with global changes in splicing regulators and splice variants driven by this process. In addition, specific alterations in AS play functionally important roles in regulating EMT at multiple levels. For example, several studies have identified EMT-specific AS signatures in breast cancer (Qiu et al., 2020; Shapiro et al., 2011). These global analyses identified widespread changes in AS events and splicing regulators in both cellular models of EMT and in patient samples, and a signature of 25 AS events that identify tumours with high levels of EMT that can robustly predict patient outcome (Qiu et al., 2020). The regulation of EMT has been explicitly linked to AS, via alterations in splicing regulators and through the function of specific AS events (Fig. 3). Examples of this will be discussed in the following section.

#### Splicing factors linked to EMT

Several studies have investigated the functional role of specific splicing factors and AS events in mediating EMT (Fig. 3A). For example, the splicing factors ESRP1, hnRNPM, SRSF1 and RBFOX2 have been associated with EMT. ESRP1 controls an epithelial-specific splicing programme and is downregulated by the EMT transcription factors Zeb1 and Snail (Horiguchi et al., 2012; Reinke et al., 2012). ESRP1 and hnRNPM were found to discordantly co-regulate changes in AS during EMT, promoting differing exon inclusion or skipping events that drove an EMT-specific splicing programme (Harvey et al., 2018). These factors' target sequences were enriched in genes associated with cell migration and cytoskeletal organisation, suggesting a direct function of ESRP1 and hnRNPM in mediating these processes. More specifically, ESRP1 promotes the inclusion of exon 4 of the Wnt transcription factor TCF4. This gives rise to an isoform with impaired Wnt transactivation activity, directly linking ESRP1 to reduced Wnt signalling, a hallmark of the epithelial cell phenotype (Weise et al., 2010). RBFOX2, the expression of which is induced upon EMT, is an important regulator of mesenchymal splicing events (Ahuja et al., 2020; Braeutigam et al., 2014; Venables et al., 2013). Interestingly, while being required for inducing a mesenchymal cell-like splicing programme, RBFOX2 is dispensable for the TGF-β-induced phenotypic changes associated with EMT (Braeutigam et al., 2014). However, depletion of RBFOX2 reduces the invasive potential of these cells, suggesting that key characteristics of invasive cancer cells depend on these splicing alterations. Another splicing regulator linked to EMT is SRSF1, an SR protein commonly upregulated in cancer and known to mediate several oncogenic processes (Oltean and Bates, 2014). SRSF1 expression is increased during EMT by AS to produce a transcript that does not undergo nonsense-mediated decay (Valacca et al., 2010). SRSF1 activity is also regulated through phosphorylation by kinases such as SRPK1 and CLK1, leading to nuclear accumulation (Aubol et al., 2018; Goncalves et al., 2014). This dual expression and localisation regulation induces specific splicing events linked to EMT. More recently, A-kinase anchor protein (AKAP8) was identified as a novel AS regulator and key driver in metastatic breast cancer through its interaction with hnRNPM. Genome-wide analysis upon AKAP8 knockdown further identified the inclusion of exon 11 of *CLSTN1* as a promoter of EMT (Hu et al., 2020). These multiple examples highlight the various mechanisms by which splicing factors regulate EMT and cellular plasticity.

Numerous studies have also linked specific AS events to critical regulatory checkpoints of the EMT process, including the induction of EMT transcription factors, the loss of cell polarity and cellular adhesion, cellular invasion and tumour–stromal signalling. Examples of how specific splice isoforms control EMT is outlined below.

#### EMT transcription factor induction

The constitutively active splice variant of RAC1, which includes exon 4 and is termed RAC1B, is overexpressed in multiple tumour types. Identified as a direct splice target of Srsf1 in a murine model of mammary carcinoma, Rac1b has been shown to mediate EMT via the production of reactive oxygen species (ROS). Increased ROS following Rac1b overexpression induces the EMT transcription factor Snail and upregulates vimentin. This in turn leads to induction of EMT, bypass of senescence and tumour development (Radisky et al., 2005).

#### Loss of cell polarity, cellular adhesion and invasion

The loss of cellular polarity and adhesion is a critical step in EMT. NUMB is a key mediator of cell polarity and adhesion via its interactions with the Par polarity complex and E-cadherin. Disruption of these interactions during EMT leads to loss of cellular adhesion and cell migration. NUMB binds these proteins through its phospho-tyrosine binding domain, which contains an epithelial-specific exon, the inclusion of which is regulated by ESRP1. Interestingly, this exon is excluded upon EMT, when ESRP1 is downregulated, thus likely disrupting these interactions and promoting the acquisition of invasive properties (Warzecha et al., 2010). One of the first identified EMT-linked AS events was the splicing of the tyrosine kinase receptor RON (also known as MST1R) by SRSF1. RON activates a signalling cascade leading to cell migration and invasion, and a constitutively active splice variant termed ΔRON, generated via SRSF1-mediated skipping of exon 11, activates these processes (Ghigna et al., 2005). ΔRON production is also controlled by the actions of other splicing regulators, including hnRNPA1 and hnRNPA2 (also known as HNRNPA2B1), which promote or suppress ΔRON production, respectively (Bonomi et al., 2013; Golan-Gerstl et al., 2011). In addition to RON splicing, the AS of CD44 has been linked to cellular invasion in multiple cancer types. CD44 has numerous splice variants, with CD44v being common in epithelial cells and CD44s expressed in mesenchymal cells (Brown et al., 2011). ESRP1 controls the shift between CD44v and CD44s isoforms and is critical for regulating EMT in both breast and ovarian cancer (Bhattacharya et al., 2018; Brown et al., 2011). Mechanistically, CD44s activates Akt signalling, increasing stem cell activity and promoting cellular invasion and chemoresistance (Bhattacharya et al., 2018; Brown et al., 2011). This isoform is also linked to high-grade human breast tumours (Brown et al., 2011), indicating a functional role in human cancer.

#### Tumour–stromal signalling

An important characteristic of EMT is the ability of cells to remodel extracellular stroma. The FGFR family of receptor tyrosine kinases are key regulators of tumour–stroma interactions, and AS can play a role in this process. AS of FGFR2 exon 3 can regulate FGFR signalling activity, and this isoform switching has been implicated in EMT in multiple cancer types (Oltean et al., 2006; Ranieri et al., 2016; Shirakihara et al., 2011; Zhao et al., 2013). Indeed, a switch from FGFR2b to FGFR2c was identified during EMT, with ESRP1 and ESRP2 believed to control this switch (Bebee et al., 2015; Warzecha et al., 2009). Mechanistically, this isoform switch promotes ERK1/2 (also known as MAPK3/MAPK1) signalling and sustains the high levels of ZEB1 expression needed to maintain a mesenchymal phenotype. Interestingly, FGFR inhibitors can restore epithelial traits by suppressing ZEB1 expression (Osada et al., 2019). Together, these examples outline the role of AS in some of the key characteristics of EMT. In addition, as alternatively spliced gene products are essential for this transition between cell states, AS may also provide opportunities for therapeutic targeting.

### Regulation of cellular metabolism

Tumour cells rely on various forms of energy for maintaining their growth. This energy can be generated by the electron transport chain in mitochondria (Raimondi et al., 2020) or via one-carbon metabolism in the mitochondrial, cytosolic and nuclear cellular compartments (Dekhne et al., 2020). Tumour growth is also fuelled by various forms of amino acid metabolism (Gu et al., 2017; Nakamura et al., 2018; Yue et al., 2017). As a key process via which tumour cells adapt to their environment, it is unsurprising that AS plays a central role in controlling cellular metabolism.

A key example of this is the role of AS in regulating the functions of two central glycolysis proteins, the glucose transporter GLUT1 (also known as SLC2A1) and pyruvate kinase isoform 2 (PKM2; also known as PKM). GLUT1-imported glucose can be metabolised to pyruvate, while PKM2 catalyses the final enzymatic reaction in the glycolytic pathway (Kozlovski et al., 2017). In HCC, tumorigenesis can be fuelled by a reliance on glycolytic energy derivation (DeWaal et al., 2018). The correct splicing of GLUT1 and PKM2 in HCC was found to be regulated by an RNA helicase, MTR4 (also known as MTREX) (Fig. 3B), which, when depleted, decreased tumour growth *in vivo*. This could be rescued by the ectopic overexpression of the fully functional isoform of GLUT1 (Yu et al., 2020). This indicates that AS is needed for efficient glucose metabolism to drive tumour growth and that it represents a potential therapeutic vulnerability.

AS can also affect mitochondrial function and oxygen metabolism, leading to increased tumour aggressiveness. Metadherin is an oncogene involved in several tumour-promoting cellular processes. A novel splice variant caused by the skipping of exon 7 (MTDHΔ7) increased NF-κB signalling to promote SOD2 expression and SIRT3 activation. These elevated the extracellular acidification and the oxygen consumption rates, which led to increased tumourigenesis (Neeli et al., 2020). Interestingly, while AS can regulate the expression of genes that affect mitochondrial metabolism, it has also been demonstrated that, reciprocally, mitochondrial content itself can affect AS (Guantes et al., 2015) – an intriguing feedback mechanism of gene regulation.

AS is also an important mediator of nutrient metabolism via the mammalian target of rapamycin complex 1 (mTORC1). mTOR is an important regulator of cell growth and modulates nucleotide, protein and lipid synthesis. The activity of mTORC1 is activated by the kinase S6K (also known as RPS6KB1), the activity of which is controlled by AS. S6K has two isoforms with opposing functions: a long one that inhibits mTORC1 and a shorter one that activates it and promotes its oncogenic function. SRSF1 controls the splicing of S6K, in turn mediating mTORC1 activity (Ben-Hur et al., 2013; Michlewski et al., 2008). Together, these examples outline the emerging functions of AS as a key mediator of cellular metabolism and highlight the potential to therapeutically target this function.

### *In vivo* and three-dimensional (3D) models to study AS

The consequences of aberrant AS in cancer have been explored in great depth using many models and techniques (Box 1). Although *in vitro* models have provided fundamental insights into potential mechanisms and therapeutic targets, the importance of the tumour microenvironment, cellular heterogeneity and cellular plasticity for tumour progression, and how AS contributes to these, may only be gleaned using more complex *in vivo* models. Such *in vivo* models may allow the study of complex genotype–phenotype relationships. Furthermore, alternatively spliced variants may only be present in a certain cell population or populations within the tumour. For example, the previously discussed Rac1, which facilitates Wnt-driven LGR5^+^ intestinal stem cell proliferation (Myant et al., 2013a,b), can be alternatively spliced into Rac1b. Rac1b was recently identified as a spliced isoform enriched in the intestinal crypt compartment. Furthermore, Rac1b is required for oncogenic Wnt signalling, as well as for the resultant tumour initiation phenotype that follows Apc loss *in vivo.* Importantly, depletion of RAC1B re-sensitised EGFR inhibitor-resistant human CRC organoids derived from liver metastases to EGFR inhibition (Gudino et al., 2021b). This demonstrates that targeting alternatively spliced isoforms can be a therapeutically viable option in metastatic CRC, and that the use of complex *in vivo* models to study these isoforms may provide fundamental insights into isoform expression in different tissue compartments.

Owing to the complexity of tumourigenic processes, the importance of using more sophisticated model systems to better recapitulate the tumour microenvironment and cellular heterogeneity cannot be overstated. This complexity can, in part, be achieved by using organoid models grown in 3D environments that mimic the extracellular matrix. Interestingly, various components of the extracellular matrix, such as collagen IV and laminin, regulate AS (Srebrow et al., 2002), and, concurrently, matrix stiffness can affect splicing factor activity (Bordeleau et al., 2015). In addition, splicing factor overexpression in 3D cultures may lead to increased cellular transformation capacity (Anczukow et al., 2012), indicating an important role for the ECM in controlling AS, and supporting the inclusion of both 3D and two-dimensional (2D) model systems in investigating splicing factors and global changes in AS. However, *in vitro* models have a number of limitations that can currently only be compensated for by using *in vivo* models.

Table 1 summarises a number of isoform-specific *in vivo* models; however, there appears to be a lack of important mouse models. It should also be noted that numerous mutant splicing factor knock-in mouse models have been developed, including the SRSF2^P95H^ (Kim et al., 2015), SF3B1^K700E^ (Mupo et al., 2017) and U2AF1^S34F^ models (Fei et al., 2018). These models recapitulate the human MDS in which these splicing factor mutations are commonly found, and are therefore valuable for the study of splicing factor mutations resulting in MDS. Interestingly, a common embryonic lethality phenotype was observed in mouse models following complete and ubiquitous knockout of many splicing factors, including SRSF1 (Xu et al., 2005), SRSF2 (Wang et al., 2001), hnRNP U (Roshon and Ruley, 2005), hnRNP C (Williamson et al., 2000) and SRp20 (also known as SRSF3) (Jumaa et al., 1999). Therefore, rather than ubiquitous knockout, conditional, tissue-specific knockout mouse models may hold more promise for the study of various splicing factors, especially given that knocking out a splicing factor in individual tissue compartments could have a less detrimental effect on tissue homeostasis. This could, in turn, allow researchers to analyse the mechanisms and consequences of AS in more detail.

**Table 1.**
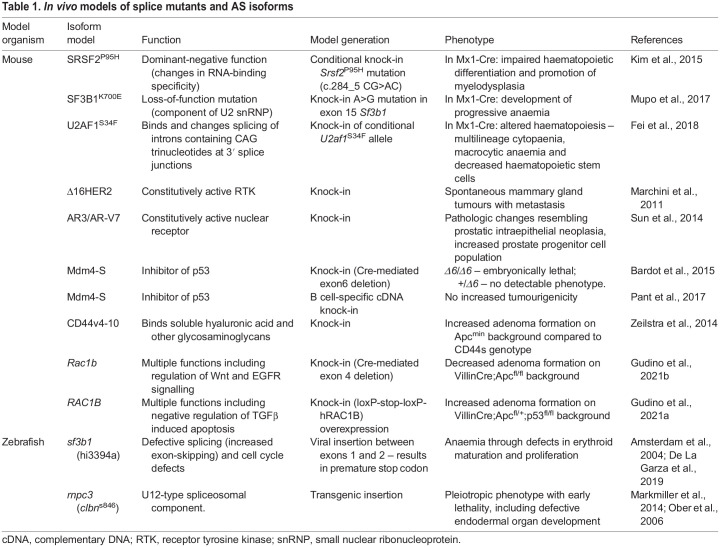
*In vivo* models of splice mutants and AS isoforms

Although these *in vivo* models have proven valuable in demonstrating phenotypes similar to human disease, there are limitations to consider when analysing global changes in AS across different model species. It is well known that AS is poorly conserved between mouse and human (Nurtdinov et al., 2003; Thanaraj et al., 2003); therefore, biomarkers or mechanisms that were originally discovered in one species may not translate well to another. It is therefore important to carefully validate changes in AS in human models, such as patient-derived organoids or patient samples, when the original identification was performed in mice or other model species. Conversely, if the same AS events are found across species, this conservation may indicate functionality. Therefore, studies utilising combinations of different model systems across multiple species are likely to identify the most important, functionally relevant AS events.

## Splicing as a therapeutic vulnerability in cancer

### AS-related therapeutic efficacy and patient prognosis

**Fig. 4.**
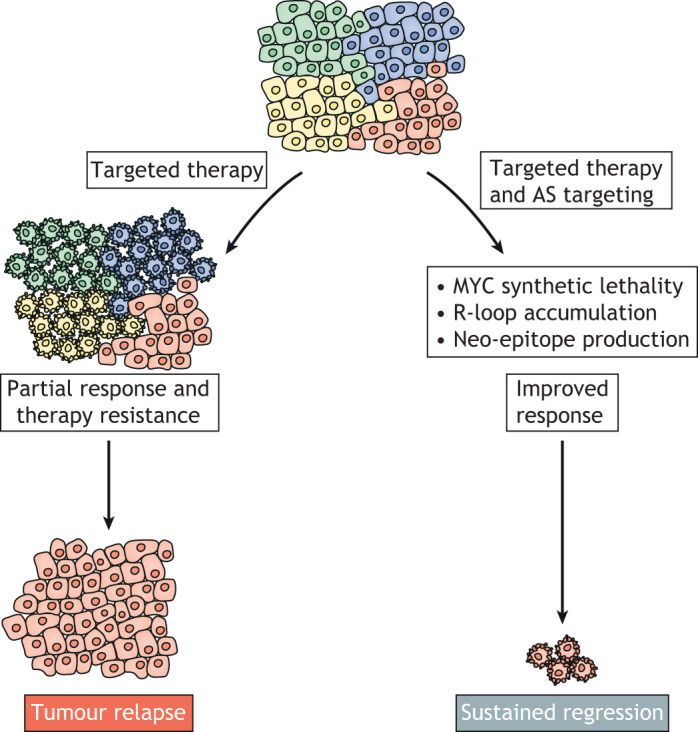
**AS as a therapeutic target.** Targeted cancer therapies often have non-durable responses, with therapy resistance and tumour relapse being common occurrences. There are multiple reasons for this, including tumour heterogeneity and intrinsic cellular plasticity. Targeting RNA splicing may lead to durable responses, as it would activate multiple anti-tumour mechanisms. These include synthetic lethality with MYC, accumulation of R-loops leading to PARP inhibitor sensitivity, and neo-epitope production leading to anti-tumour immunity and enhanced efficacy of immunotherapies. In addition, as AS is a core cellular process, it is likely that resistance to its targeting will be less likely to arise due to limited bypass mechanisms.

As cancer is a complex, heterogeneous disease, treating it is extremely challenging, with the emergence of resistance being a common occurrence. In particular, despite huge promise, highly specific targeted therapies against single oncogenic alterations often give limited patient benefit. Patient stratification can increase the effectiveness of such therapies, but limits the number of patients who benefit from them. In addition, although immunotherapies have shown striking effectiveness, their use is limited to certain patient subgroups with high mutational burden. Therefore, there is a pressing need to develop new therapeutic strategies, either by identifying targets less prone to the emergence of resistance, or by finding ways to improve the efficacy of commonly used targeted and immune therapies. In this section, we will discuss the potential of targeting AS and how this might produce durable anti-cancer responses and improve the effectiveness of current therapies (Fig. 4).

Mutations in and varying expression levels of splicing factors can result in significantly different patient prognoses, as well as in varied response to therapeutic intervention (Bonnal et al., 2020). Changes in the expression of alternatively spliced isoforms can confer sensitivity or resistance to therapeutics and varied prognoses, depending on which tumoural or stromal isoform is present (Hagiwara et al., 2020; Wang et al., 2016b). Therefore, a concerted effort is currently underway to develop therapies targeted towards mutant splicing factors and overexpressed spliced isoforms, which may both confer either sensitivity or resistance. For more detail, we direct the readers to a recent comprehensive review on therapeutic modulation of splicing (El Marabti and Abdel-Wahab, 2021).

As mentioned previously, SF3B1 is a core component of the U2 snRNP spliceosome complex (Zhang et al., 2020). Mutations in *SF3B1* are found predominately in blood cancers, including chronic lymphocytic leukaemia (Liu et al., 2020), MDS (Singh et al., 2020), and both secondary and *de novo* acute myeloid leukaemia (AML) (Bamopoulos et al., 2020), but also in solid tumours, including melanomas (Nassar and Tan, 2020) and breast cancer (Fu et al., 2017). Patients with *SF3B1* mutations tend to have shorter overall survival (Bonnal et al., 2020). Mutant SF3B1 can be targeted by the macrocyclic lactone Pladienolide B. Its derivatives, including E7107 and H3B-8800, have demonstrated effectiveness against various mutant splicing factors, and H3B-8800 is currently undergoing a first-in-human Phase I clinical trial for the treatment of chronic myelomonocytic leukaemia, AML and MDS. Interestingly, whereas SF3B1^K700E^ and SRSF2^P95H^ mutations cause sensitivity to H3B-8800 and E7107 (Obeng et al., 2016; Seiler et al., 2018b), the SF3B1^R1074H^ mutation can result in resistance to the same two compounds (Teng et al., 2017; Yokoi et al., 2011). These results highlight the need for careful genomic stratification of patients with splicing factor mutations before therapeutic intervention.

Alternatively spliced isoforms of known oncogenic drivers also drive resistance to targeted therapies. For example, the BRAF^V600E^ mutation constitutively activates the MAPK pathway and primes oncogenesis in many cancers, including melanoma and CRC. Targeted therapeutics such as vemurafenib have demonstrated clinical efficacy for many patients harbouring these mutations, although resistance to vemurafenib is prevalent. An alternatively spliced isoform of BRAF (BRAF3-9) excludes the RAS-binding domain and thus mediates resistance to vemurafenib in *in vitro* and *in vivo* melanoma models. Overcoming vemurafenib resistance in melanoma cell lines expressing high levels of BRAF3-9 required treatment with the SF3B1 inhibitor spliceostatin A, which reduced BRAF3-9 levels and subsequently downregulated ERK signalling (Salton et al., 2015). Furthermore, AS has been implicated as a mediator of synthetic lethality in MYC-driven cancers. Targeting both core spliceosomal components in MYC-dependent breast cancer (Hsu et al., 2015) and snRNP assembly through PRMT5 in MYC-driven lymphoma (Koh et al., 2015) impaired tumourigenicity and implied a therapeutic vulnerability in cancers reliant on oncogenic MYC (Fig. 4). Importantly, as AS is a core cellular process, it is less clear how tumour cells could bypass their requirement for it than, for example, the activation of an oncogenic pathway, where multiple stimuli can maintain the pathway. In addition, MYC pathway activation is an extremely common cancer-driving event, suggesting that AS-mediated synthetic lethality may impart therapeutic vulnerability to a large number of cancer types.

Targeting AS can also have profound consequences for the maintenance of genome integrity. When dysregulated, this process can result in tumour initiation and drive tumour progression, but can also cause cancer cell death (Jeggo et al., 2016). The primary sources of threats to genome stability and integrity are DNA recombination events and transcription-associated processes. The latter can result in the formation of RNA:DNA hybrids known as R-loops, whereby nascent RNA can hybridise to template DNA and displace single-stranded DNA (ssDNA) (Aguilera and Gaillard, 2014). R-loops can cause genome instability by disturbing replication fork progression and by insults to the ssDNA from endogenous mutagenic deaminases and chemicals such as ROS (Su and Freudenreich, 2017; Tan et al., 2020b). Therefore, many splicing-related proteins, including SR and SF3B proteins that regulate these transcription-associated processes are critical in preventing the accumulation of R-loops and maintaining genome stability. Recently, Chen et al. described the link between mutations in SR proteins leading to R-loop accumulation and MDS, whereby SRSF2^P95H^ and U2AF35^S34F/Q157P^, both of which drive global changes in exon-skipping events, also increased R-loop-associated mutagenesis. Overexpression of RNASEH1, an endonuclease that specifically degrades the RNA in RNA:DNA hybrids, rescued the phenotype of the mutant haematopoietic progenitors to resemble that of wild-type cells (Chen et al., 2018). Furthermore, a study has shown that widespread R-loop-associated chromosomal instability occurs in embryonal tumours with multi-layered rosettes due to various germline and somatic mutations in the endoribonuclease DICER1 (Lambo et al., 2019). Interestingly, the authors of this study demonstrate that R-loops may be targeted therapeutically by poly(ADP-ribose) polymerase 1 and topoisomerase 1 inhibition. These results raise an interesting question of whether application of these targeted therapies may have a wider scope in malignancies driven by R-loops, and whether these can be applied to tumours in which dysfunctional SR protein-mediated R-loop formation is a primary driver of tumourigenesis.

#### Targeting splicing to mediate cellular growth and cell death

Tissue homeostasis depends on many factors, one of the most important being the equilibrium between cell growth and death. Therefore, dysregulation of this balancing act can result in many pathologies, including cancer (Hanahan and Weinberg, 2011). Many different mechanisms of cell death exist, including the classical intrinsic and extrinsic apoptotic pathways and more recently defined ones, such as ferroptosis (Galluzzi et al., 2018). The classical extrinsic apoptotic pathway can be activated through extracellular sources, primarily through the FAS (CD95) receptor after binding of an apoptosis-initiating ligand (e.g. FAS ligand), which results in proteolytic cleavage of procaspases and subsequent apoptosis. The intrinsic pathway is induced by intracellular sources, such as nutrient deprivation and DNA damage, and signals through pro- and anti-apoptotic mitochondrial proteins, including Bcl-2, Bcl-X_L_ and Bax/Bak (also known as BAK1) (Pohl et al., 2018). The AS of many of these proteins has been implicated in cancer cell survival and therapeutic resistance (Paronetto et al., 2016). For example, the *BCL2L1* gene can undergo AS via an alternative 5′ splice site on exon 2, yielding two isoforms – the long isoform Bcl-X_L_ and the short isoform Bcl-X_S_ (Stevens and Oltean, 2019). Bcl-X_L_ contains BH1, 2, 3, 4 domains while Bcl-X_S_ contains only BH3, 4. Importantly, the BH1, 2 domains act as the anti-apoptotic regulators, sequestering pro-death proteins in the mitochondrial membrane to prevent outer membrane permeabilisation (Shimizu et al., 2000). Therefore, Bcl-X_L_ and Bcl-X_S_ are considered anti- and pro-apoptotic, respectively. Therapeutic targeting efforts include the development of small-molecule inhibitors of anti-apoptotic splice variants, such as Bcl-X_L_ (Pohl et al., 2018), and the design of antisense oligonucleotides to skew AS in favour of the pro-apoptotic Bcl-X_S_ isoform (Bauman et al., 2010; Mercatante et al., 2002). Furthermore, alternatively spliced isoforms of pro-apoptotic proteins have also been linked to therapy resistance. Imatinib, a tyrosine kinase inhibitor of BCR-ABL in CML, can also bind to the BH3 domain of the Bcl-2 family member BIM (also known as BCL2L11), promoting its pro-apoptotic function (Kuroda et al., 2006). AS resulting in the skipping of exon 4 promotes the BIM-γ isoform, which lacks the BH3 domain and therefore mediates resistance to Imatinib (Isobe et al., 2016). Given the prominent role of cell death dysregulation in cancer, further exploration into how AS may drive carcinogenesis and therapeutic resistance in the newly discovered pathways, such as ferroptosis, may provide important insights into novel mechanisms that may offer therapeutically targetable options.

#### AS in immunotherapy and immune evasion

**Fig. 5.**
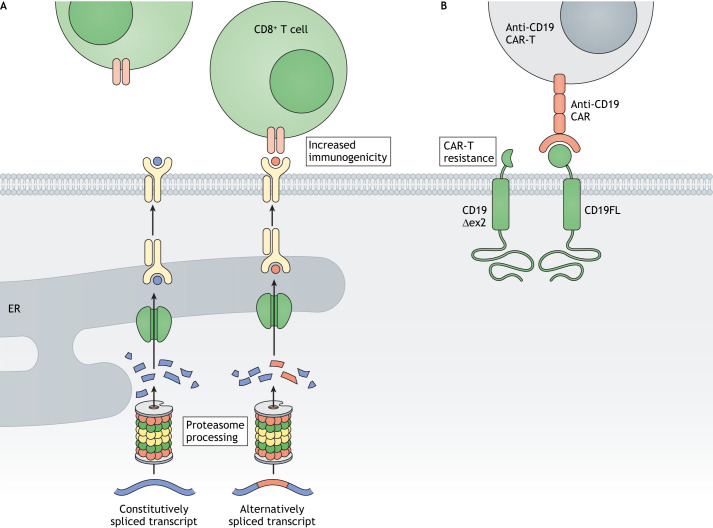
**AS splicing in cancer neoantigen generation and immunotherapy.** (A) AS variants can produce neoantigens. Following proteosomal processing, these novel peptides from AS variants are transported into the ER, where they are bound by MHC Class 1 molecules and secreted to the cell surface via Golgi-mediated transport. Once on the surface, these neoantigens elicit an increased CD8^+^ T-cell response. However, this anti-tumour immune response is typically hampered by the presence of the immune checkpoint. (B) CD19 is a cell surface antigen that is a suitable target for cell-based immunotherapy with CAR-Ts. An alternatively spliced variant of CD19 lacking exon 2 is missing part of its extracellular domain and retains CD19 in the cytoplasm. This is believed to make CD19Δex2-expressing cancer cells resistant to anti-CD19 CAR-T-targeted therapy. CD19, B-lymphocyte antigen 19; CAR-T, chimeric antigen receptor T cell; ER, endoplasmic reticulum; MHC, major histocompatibility complex.

The discovery of the immune checkpoint proteins CTLA-4 (Leach et al., 1996) and PD-1 (also known as PDCD1) (Ishida et al., 1992) has revolutionised our understanding of immunity in cancer and has resulted in the introduction of therapeutic vaccines, immune checkpoint inhibitors, T-cell receptor-engineered T cells and chimeric antigen receptor T cells (CAR-Ts). These have ushered in new personalised medicine approaches for both blood and solid cancers. Host T cells can bind cancer-derived antigenic peptides, mounting an immune response against the cancer. Termed neoantigens, the most commonly studied sources of these peptides are non-synonymous DNA mutations, cancer germline antigens and antigens derived from viral oncogenes (Frankiw et al., 2019). The efficacy of immunotherapies relies on the selection of appropriate cancer-specific antigens, and therefore novel sources of neoantigens are highly sought after. As it is well documented that AS is dysregulated in cancer (Kahles et al., 2018), it has recently been postulated that AS may generate neoantigens, which can be targeted for immunotherapy. More specifically, the common tumour-associated AS event of intron retention (Fig. 1) has been demonstrated to produce novel peptides presented on the major histocompatibility complex-1 (Pearson et al., 2016; Smart et al., 2018), suggesting new targets for immunotherapy. Furthermore, a systematic analysis of samples from a large cancer dataset showed that splice-site mutations doubled the amount of neoantigens, and these were more immunogenic than those generated from other non-synonymous mutations. Splice-site mutations were also found to occur in the known cancer driver genes *TP53*, *PTEN* and *BRCA1* (Jayasinghe et al., 2018). More recently, it has been demonstrated that pharmacological modulation of AS leads to the production of neoantigens, elicits anti-tumour immunity and enhances the efficacy of immune checkpoint inhibitors (Lu et al., 2021). Interestingly, these findings were consistent across multiple different inhibitors of AS, suggesting that perturbed splicing is a rich source of neoantigen production (Fig. 5A). Indeed, perturbing splicing generated over 100 neoantigens that were able to trigger an endogenous T-cell response. This suggests that RNA splicing modulation produces splicing-specific neoantigens with potential for enhancing the clinical efficacy of immunotherapy (Fig. 4). Lastly, while AS can benefit immunotherapy, it has also been implicated in resistance against it. CD19, a target for CAR-T therapy in B-cell acute lymphoblastic leukaemia, is alternatively spliced into various isoforms, including one generated by skipping of exon 2. This isoform is retained in the cytoplasm and is masked from T-cells, therefore providing a resistance mechanism against CART-19 therapies (Fig. 5B) (Sotillo et al., 2015). These examples provide evidence of a ‘double-edged sword’ role of AS in immunotherapy.

## Conclusions and future directions

AS is a fundamental process, essential for creating proteomic and transcriptomic diversity. This has been critical in providing an evolutionary advantage and has been seen as a successful process in many organisms (Keren et al., 2010). When put in the context of cancer, AS can provide the same advantages, contributing to tumour heterogeneity, disease progression and drug resistance. Some important unanswered questions remain central to AS in less-understood processes, such as tumour heterogeneity and cellular plasticity. For example, does RNA splicing contribute to the heterogeneous response of tumours to therapy? Can targeting it enhance therapy effectiveness? Can the ability of cells to trans-differentiate and de-differentiate into stem cells be perturbed by inhibiting transcriptome diversity mediated be AS? And finally, can RNA splicing modulation be exploited to enhance the production of neoantigens, thus increasing the efficacy of immunotherapies? The targeting of mutant splicing factors has had some success, and with the increased availability and decreased cost of next-generation sequencing, careful molecular stratification should ensure an increased effectiveness of therapeutics given to patients. Furthermore, although there has been a focus on mutated splicing factors, it is now widely accepted that overexpressed splicing factors may provide good therapeutic targets as well. These splicing factors produce multiple oncogenic isoforms; therefore, instead of targeting the isoforms themselves, hitting their upstream regulators may lead to more durable responses. Our understanding of AS has advanced significantly, and it is encouraging that these advances are already being translated into successful therapies for cancer.
